# QUDeX-MS: hydrogen/deuterium exchange calculation for mass spectra with resolved isotopic fine structure

**DOI:** 10.1186/s12859-014-0403-1

**Published:** 2014-12-11

**Authors:** Joseph P Salisbury, Qian Liu, Jeffrey N Agar

**Affiliations:** Departments of Chemistry and Chemical Biology and Pharmaceutical Sciences and Barnett Institute of Chemical and Biological Analysis, Northeastern University, 360 Huntington Avenue, Boston, MA 02115 USA

**Keywords:** Hydrogen/deuterium exchange, Isotopic fine structure, Fourier transform ion cyclotron resonance, Mass spectrometry, Protein structure, Protein analysis

## Abstract

**Background:**

Hydrogen/deuterium exchange (HDX) coupled to mass spectrometry permits analysis of structure, dynamics, and molecular interactions of proteins. HDX mass spectrometry is confounded by deuterium exchange-associated peaks overlapping with peaks of heavy, natural abundance isotopes, such as carbon-13. Recent studies demonstrated that high-performance mass spectrometers could resolve isotopic fine structure and eliminate this peak overlap, allowing direct detection and quantification of deuterium incorporation.

**Results:**

Here, we present a graphical tool that allows for a rapid and automated estimation of deuterium incorporation from a spectrum with isotopic fine structure. Given a peptide sequence (or elemental formula) and charge state, the mass-to-charge ratios of deuterium-associated peaks of the specified ion is determined. Intensities of peaks in an experimental mass spectrum within bins corresponding to these values are used to determine the distribution of deuterium incorporated. A theoretical spectrum can then be calculated based on the estimated distribution of deuterium exchange to confirm interpretation of the spectrum. Deuterium incorporation can also be detected for ion signals without a priori specification of an elemental formula, permitting detection of exchange in complex samples of unidentified material such as natural organic matter. A tool is also incorporated into QUDeX-MS to help in assigning ion signals from peptides arising from enzymatic digestion of proteins. MATLAB-deployable and standalone versions are available for academic use at qudex-ms.sourceforge.net and agarlabs.com.

**Conclusion:**

Isotopic fine structure HDX-MS offers the potential to increase sequence coverage of proteins being analyzed through mass accuracy and deconvolution of overlapping ion signals. As previously demonstrated, however, the data analysis workflow for HDX-MS data with resolved isotopic fine structure is distinct. QUDeX-MS we hope will aid in the adoption of isotopic fine structure HDX-MS by providing an intuitive workflow and interface for data analysis.

## Background

Incubation of molecules in deuterium oxide and measurement of exchange between hydrogen and deuterium (i.e. hydrogen/deuterium exchange, abbreviated as HDX) is a versatile technique for characterizing molecular structure and reactivity. Coupled with mass spectrometry (HDX MS), the development and application of HDX MS to the study of proteins has led to a diverse range of methodologies for the characterization of protein structural dynamics, stability, and molecular interactions. The versatility of HDX MS has been demonstrated through a range of analytical applications including studying folding/unfolding [1-3] and oligomerization [4-8]; how mutation [9-12], posttranslational modification [13-17], and interaction with other molecules affect protein structures [18-25]; and the quality of biopharmaceuticals [26-28]. In contrast to other techniques capable of mapping protein dynamics in solution such as nuclear magnetic resonance, HDX MS consumes far less sample, requires lower concentrations, and can analyze larger protein complexes. In a typical HDX MS workflow examining protein structure, proteins are labeled with deuterium oxide around physiological pH for a range of periods of time (from seconds to days), followed by a reaction quenching including a drop to acidic pH (~2.5) and lowered temperature (0°C) [29]. To map deuterium incorporation, and in turn, map extent of solvent exposure of residues, a bottom-up strategy is often adopted whereby proteases (capable of digesting proteins under the acidic quench conditions) break down proteins prior to MS analysis. MS analysis is often performed by electrospray ionization (ESI), although matrix-assisted laser desorption/ionization (MALDI) can also be beneficial [30,31]. Liquid chromatography (LC) is often utilized prior to MS to separate digest peptides and increase protein coverage. A number of computational utilities have been introduced to streamline the analysis of HDX MS data, including HDX-Analyzer [32], HX-Express [33], Deuterator [34], HD Desktop [35], DEX [36], Hydra [37], TOF2H [38], and Hexicon [39,40].

**Figure 1:**
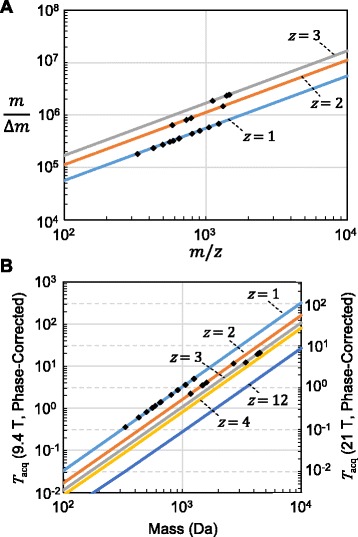
**Resolving power and acquisition time required to observe deuterium-associated isotopic fine structure is charge state dependent. A**. In order to resolve deuterium-associated peaks, it is necessary to detect a mass difference *m*_HDX_ of ~2.922 mDa, where *m*_HDX_ is the mass difference between ^2^H and ^1^H minus the mass difference between ^13^C and ^12^C. Increasing charge states require higher resolving power at a given *m/z*. Plotted are the resolving powers (*m*/∆*m*_50%_ where ∆m_50%_ is the full width at half-maximum peak height) required to observe deuterium-associated isotopic fine structure for *z* = 1, 2, and 3 (Eq. 2). ∆m_50%_ was calculated assuming two peaks separated by *m*_HDX_/*z* of equal height and Lorentzian peak shape are resolved with a valley between them reaches half-maximum height for either peak (Eq. 3). Also plotted (in black diamonds) are the resolving powers required to observed various peptide ions we previously monitored in a HDX experiment [9] to help illustrate the range of resolving powers necessary for one representative set of data. **B**. Theoretical minimum acquisition time required in order to resolve deuterium-associated isotopic fine structure as a function of absolute mass for various charge states. For a 9.4 T FTICR-MS operating in absorption mode, we assumed a conservative ~1.45-fold improvement in resolving power over the magnitude mode low-pressure limit (Eq. 1, effective field *B*_0_ = 13.6 T [47]). For the 12 T FTICR-MS operating in absorption mode, we assumed the maximum 2-fold improvement in resolving power over the magnitude mode low-pressure limit, which can be valid for acquisition times of less than a few seconds [46,47]. Again plotted (in black diamonds) are peptide ions monitored from an actual HDX experiment [9] to help illustrate the range of acquisition times that would be necessary without further optimization.

One caveat of HDX MS data analysis is that at typical resolving powers (including relatively high resolving powers routinely obtained on FT-Orbitrap instruments), peptide deuteration is detected as a convolution of deuterated peaks with peaks corresponding to the natural abundance distribution of heavy isotopes (i.e. ^13^C, ^15^ N, ^18^O, and ^34^S/^36^S). Thus, in order to estimate the distribution of deuterium incorporation, typical HDX analysis determines the mass centroid shift between the deuterated and the native isotope pattern of a peptide to measure average deuterium incorporation. Methods for estimating the full distribution of deuterium exchange, to detect potential bimodal exchange behaviors, have also be devised [39,40], although these methods are dependent upon sequence information and/or the undeuterated isotope distribution. To minimize back-exchange during chromatography, fast gradients are necessary, increasing the potential for overlapping peak distributions in spectra. High mass resolution can help to alleviate this problem, and algorithms have been developed to estimate accurate deuterium content in spectra with overlapping distributions at low signal-to-noise [41]. At sufficiently high resolving powers (*m*/∆*m*_50%_ ≥ 100,000), elemental compositions of peptides can be used to reliably assign overlapping distributions in a rapid and automated procedure [42]. However, as resolving power increases, the mass defect associated with different atomic nuclei binding energies leads to the detection to the isotopic fine structure. Indeed, at ultrahigh resolving powers (Figure 1A) deuterium exchange-associated peaks become distinct from natural abundance heavy isotopic species, enabling a more direct detection of the distribution of deuterium incorporation [43].

In the case of Fourier transform ion cyclotron resonance (FTICR) mass spectrometers operating at low-pressure, resolving power *m*/∆*m*_50%_ (where ∆*m*_50%_ is the full width at half-maximum peak height) at a particular *m/*z is limited in magnitude mode by the strength of the magnetic field (*B*_0_) and the time-domain ICR signal acquisition period (*T*_acq_) [44,45]:

1 $$ {\frac{m}{\mathrm{\Delta} m}}_{50\%}=\frac{1.274\times {10}^7\ z\ {B}_0\ {T}_{acq}}{m} $$

While long acquisition periods can be used to produce spectra of sufficient quality to resolve deuterium exchange-associated peaks, limiting the acquisition period is necessary in order to accurately detect these peaks on the fast LC-time scale used during HDX experiments. Increasingly powerful magnetic fields provide one means of lowering acquisition time, with fields now up to 21 T [46], although this involves substantial investment. Solving the phase of the Fourier transform spectrum in absorption mode can further increase resolving power up to 2-fold beyond the magnitude mode limit [47-50]. As deuterium-associated peaks at a given charge state are separated by a constant *m*_HDX_/*z*, where *m*_HDX_ is the mass difference between ^2^H and ^1^H minus the mass difference between ^13^C and ^12^C, the acquisition period necessary to resolve deuterium-associated peaks of a given absolute mass *M* can be estimated using:

2 $$ {T}_{acq}=\frac{{\left[M+z\ {m}_{adduct}\right]}^2}{1.274\times {10}^7\mathrm{\Delta} {m}_{50\%}\ {z}^2\ {\overline{B}}_0} $$

where ${\overline{B}}_0$ is the effective field strength in absorption mode, *m*_*adduct*_ is the ionization adduct mass (typically proton mass = ~1.00728 Da) and

3 $$ \mathrm{\Delta} {m}_{50\%}=\frac{m_{HDX}}{z}\ \frac{\sqrt{\sqrt{33}-5}}{\sqrt{2}} $$

is the peak full-width at half height necessary to resolve Lorentzian-shaped peaks of equal height separated by *m*_HDX_/*z* with valley between peaks of 50% maximum height. Thus, in absorption mode at 21 T, <1 sec acquisition periods are required to resolve deuterium exchange-associated peaks on digest peptides less than 1 kDa (Figure 1B). Indeed, for peptides less than 1 kDa, depending on charge state, absorption mode can also yield improvements significant enough to make deuterium-exchange associated peaks resolvable on the more common 9.4 T FTICR with acquisition periods of <1 sec. On high performing Orbitrap instruments, resolving powers in excess of 1 M can be achieved under certain conditions with acquisition times on the time scale of a few seconds [51], with resolving powers ~240,000 at *m/z* 400 sufficient to resolve small enough peptides with less than one second acquisition times [52]. Thus, with small enough digest fragments, HDX can be monitored based on resolved isotopic fine structure on a UPLC-time scale. However, in monitoring deuterium incorporation in this fashion, a novel data analysis workflow is necessary to take maximum advantage of this ultrahigh mass resolution data. As yet, no computational utility for this workflow exists.

Here, we present QUDeX-MS (*q*uick *u*ltrahigh-resolution hydrogen/*d*euterium *ex*change *m*ass *s*pectrometry), which was developed in order to facilitate the analysis of isotopic fine structure-resolved HDX MS data. The graphical interface, launched either as a stand-alone program or within MATLAB, guides users through the process of interpreting spectra obtained from peptides, proteins, and other molecules, providing the user with the distribution of deuterium incorporated as output.

## Implementation

**Figure 2:**
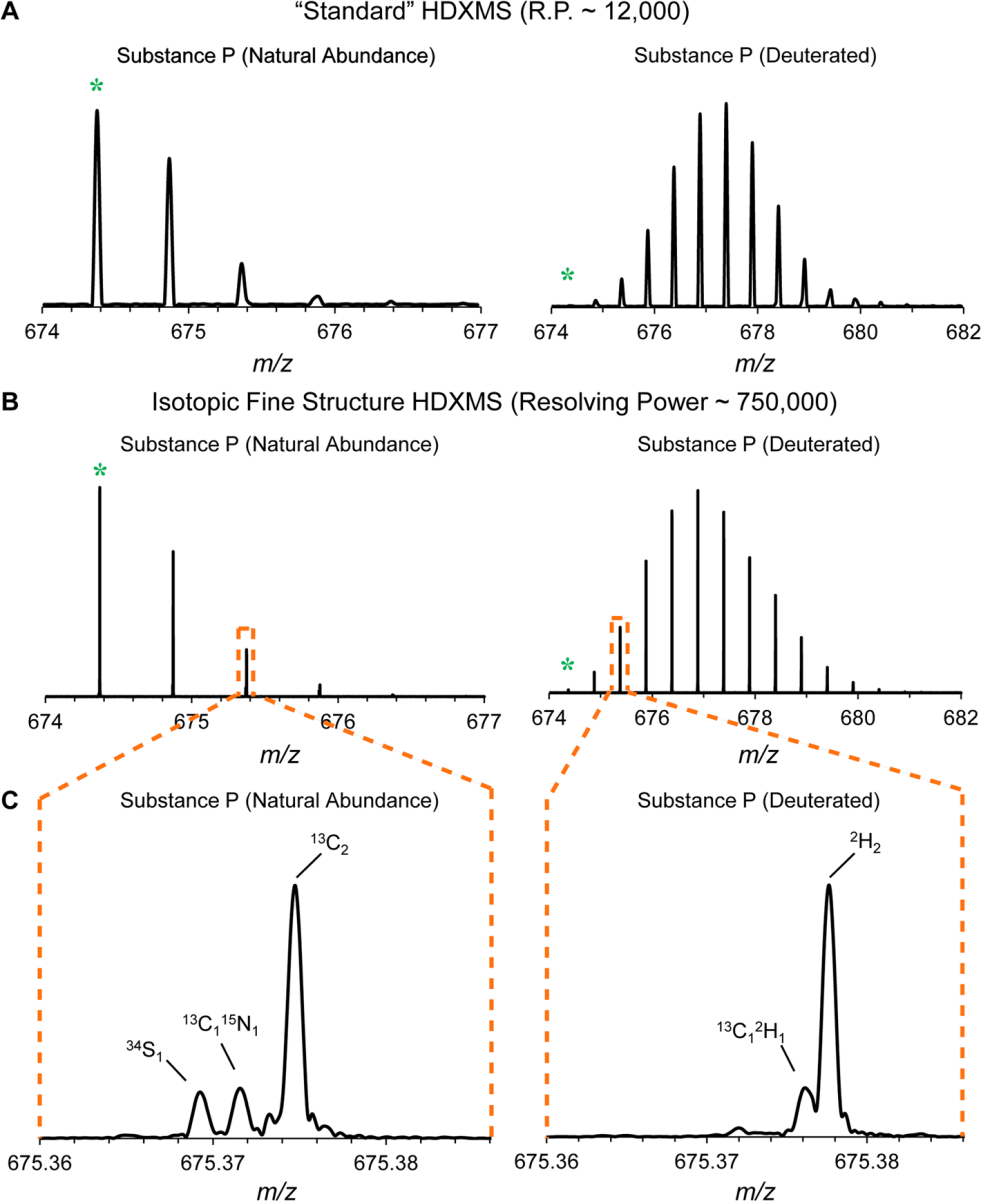
**Traditional versus isotopic fine structure HDX-MS of the [M + 2H]** ^**2+**^ **of substance P. A**. Isotope distributions of deuterated peptides (right) are shifted to heavier masses relative to the non-deuterated peptide (left). The monoisotopic isotopomer (green stars) appears depleted in the deuterated spectrum as it becomes labeled and consequently redistributed throughout the distribution. At typical resolving powers, natural abundance isotopomers and the isotopomers resulting from HDX that share the same nominal mass are not distinguished. **B**, **C**. In isotopic distributions where fine structure is resolved, deuterium-associated peaks (“pseudomonoisotopic” *m/z*’s corresponding to incorporation of 1, 2, or 3 deuterium are thus *m/z* 674.8745, *m/z* 675.3776, and *m/z* 675.8808, respectively) are distinct from the peaks corresponding to the presence of natural abundance isotopes such as the [M + 2H]^2+^ of substance P with exactly two ^13^C, which is present at *m/z* 675.3747.

For a given charge state of an ion, the distance between deuterium exchange-associated peaks in an isotopically resolved distribution is constant (the mass difference between ^2^H and ^1^H divided by charge). Thus, starting from the monoisotopic *m/z*, the *m/z*’s associated with deuterium-incorporated forms of the natural abundance monoisotopic molecule, the “pseudomonoisotopic peaks” [43], can be calculated. For example, in the case of human substance P (C-terminally amidated RPKPQQFFGLM), the monoisotopic [M + 2H]^2+^ is found at m/z 674.3713 and the pseudomonoisotopic m/z’s corresponding to incorporation of 1, 2, or 3 deuterium are *m/z* 674.8745, *m/z* 675.3776, and *m/z* 675.8808, respectively (Figure 2A). In isotopic distributions where fine structure is resolved, these deuterium-associated peaks are distinct from the peaks corresponding to the presence of natural abundance isotopes such as the [M + 2H]^2+^ of substance P with exactly two 13C at *m/z* 675.3747, substance P with exactly one ^13^C and one deuterium at *m/z* 675.3762, and substance P with exact two deuterium at *m/z* 675.3776 (Figure 2B-C).

**Figure 3:**
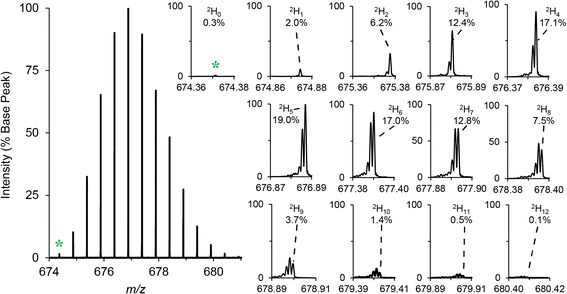
**Example calculation of deuterium incorporation based on deuterated substance P.** The [M + 2H]^2+^ of substance P is shown on the left, with each inset showing a zoomed view of the isotopic fine structure detected for each peak that is typically convoluted at lower resolving powers. QUDeX-MS determines deuterium incorporation by first finding the percent contribution each pseudomonoisotopic peak has relative to the sum of all the pseudomonoisotopic peaks plus the monoisotopic peak (green star). The average number of deuterium is then determined by multiplying these percentages by the corresponding number of deuterium incorporated for each peak. As a deuterium nuclei is approximately 0.003 Da heavier than a ^13^C nuclei, the pseudomonoisotopic peak (that is, the peak associated with deuterium being the only heavy isotope in an ion) is seen to be the rightmost peak within a fine isotopic distribution for a given nominal mass. A major contribution to the intensity of the peaks to the left of the pseudomonoisotopic peak (seen spaced 0.0015 *m/z* apart in this example, as the [M + 2H]^2+^ of substance P is shown) are the isotopomers consisting of increasing number of ^13^C and decreasing number of deuterium.

QUDeX-MS determines deuterium incorporation by first finding the percent contribution each pseudomonoisotopic peak has relative to the sum of all the pseudomonoisotopic peaks plus the monoisotopic peak (Figure 3). The average number of deuterium is then determined by multiplying these percentages by the corresponding number of deuterium incorporated for each pseudomonoisotopic *m/z*. For each of these peaks, the relative distribution of deuterium-associated peak intensities is found, and from that the mean number of deuteriums incorporated is calculated [43]. In addition to estimating the number of deuterium incorporated into the natural abundance monoisotopic species, this process is repeated for each natural abundance isotopomer that consists of all the most abundant naturally occurring stable isotopes (i.e. ^14^ N, ^16^O, and ^32^S) except for an increasing number of ^13^C (i.e. the isotopomers corresponding to one, two, etc. ^13^C and no other heavy atoms). A final estimated mean number of deuterium incorporated is calculated based on the most abundant of the ^13^C-associated isotopomer distributions, which is often the monoisotopic (^13^C = 0) species for smaller peptides.

In order to calculate the distribution of deuterium incorporated in a given molecule such as a peptide or protein, a spectrum to be analyzed can be loaded in QUDeX-MS. To demonstrate usage of QUDeX-MS, a spectrum of deuterated substance P is provided as sample data with the installation package. Spectra to be analyzed should be formatted as a two-column (*m*/*z* and corresponding intensity) ASCII file with no headers and standard column delimiters (i.e. whitespace or commas). Spectra can be denoised using wavelets [53-55] and baseline subtracted within QUDeX-MS if necessary using the included Preprocessing Tool. Adjustments can be made to slide the *m/z* alignment within QUDeX-MS, but if proper assignments are to be made to a reference sequence or formula, it is necessary the spectra be well-calibrated beforehand. If necessary, a table of deuterium-associated *m/z*’s (based on a specified monoisotopic mass and charge state) can be exported from QUDeX and used to calibrate spectra in another program prior to loading into QUDeX.

**Figure 4:**
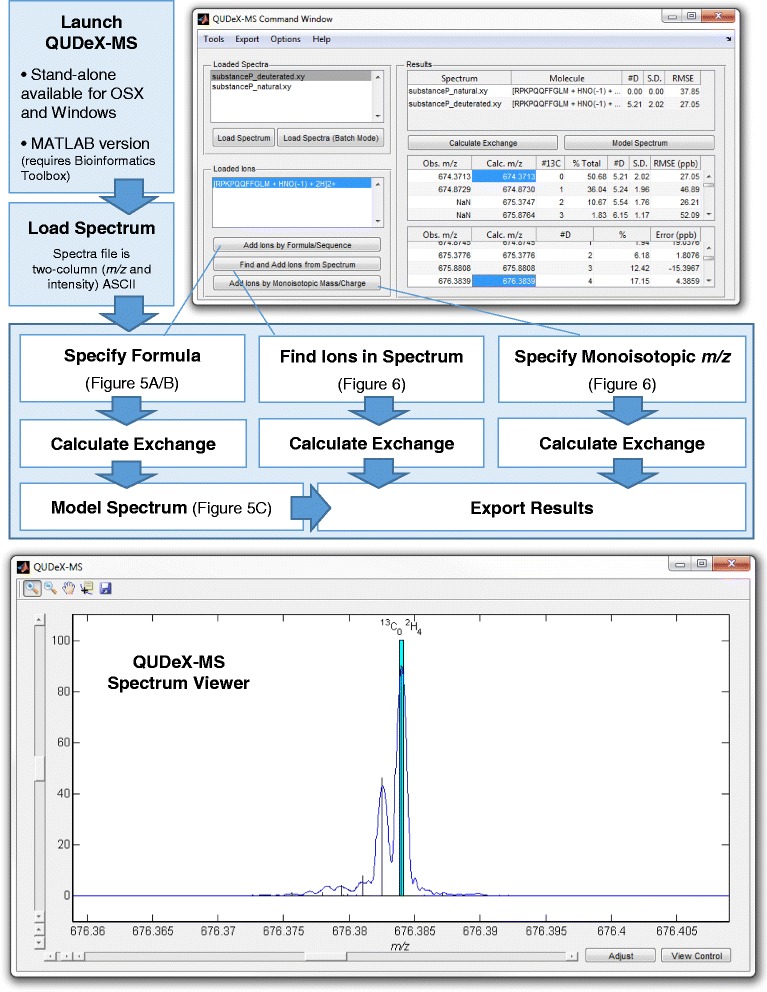
**Overview of QUDeX-MS possible workflows.** QUDeX-MS can be launched either from MATLAB or as a standalone executable. Once loaded, a *Command Window* (top right) and *Spectrum View* window (bottom) appear. Spectra can be loaded one at a time, with all loaded spectra managed in the *Loaded Spectra* listbox. The active spectrum selected in the *Loaded Spectra* listbox will be displayed in the *Spectrum Viewer* window. After loading a spectrum to analyze, several routes can be chosen for estimating deuterium incorporation of ion signals in the spectrum. An ion can be selected for analysis either by specifying a formula (or peptide sequence), locating a distribution in the spectrum, or specifying a monoisotopic *m/z*. Once an ion is loaded, it is displayed in the *Loaded Ion* listbox, and a deuterium incorporation distribution can be determined from the selected spectrum by selecting *Calculate Exchange*. Specific deuterium-associated peaks can be selected from the *Command Window* to quickly navigate to a zoomed view of their region of the spectrum in the *Spectrum Viewer*. If the elemental formula has been specified, the distribution of deuterium incorporation can then be used to calculate a theoretical spectrum by selecting *Model Spectrum*. A calculated isotopic fine structure line spectrum will then be displayed over the loaded spectrum in the *Spectrum Viewer* window.

After loading an experimental spectrum to analyze, several routes can be chosen for estimating deuterium incorporation of ion signals in the spectrum (Figure 4). If the exact elemental formula (or amino acid sequence) of the ion signal to be analyzed is known, this can be provided to QUDeX-MS. If the elemental composition of an ion signal being analyzed is not known, or if the user does not want to specify it a priori, deuterium incorporation can be estimated by detecting isotopically resolved ion signals in the spectrum within QUDeX-MS, or by simply specifying a monoisotopic mass from which to calculate a deuterium-associated *m*/*z* distribution.

The precise location of the deuterium-associated peaks for a given natural abundance isotopomer are then projected on the spectrum. The *m/z* alignment (mass calibration) of the spectrum can be adjusted, if necessary, to better fit with the calculated deuterium-associated peaks. Once the user is satisfied with the alignment, the distribution of deuterium incorporation can be determined from the spectrum by QUDeX-MS. If the elemental formula has been specified, the distribution of deuterium incorporation can then be used to calculate a theoretical line spectrum, which is projected onto the loaded spectrum. Calculated theoretical spectra based on estimated deuterium incorporation can also be exported as two-column ASCII files for additional analyses.

For automated processing, spectra can be batched loaded using a simple configuration file (sample file and instructions included with QUDeX-MS releases). If the sequences or formulas are known for spectra being loaded in batch, this can also be specified in the configuration file, and spectra will be processed automatically. A digest tool is provided with QUDeX-MS that can be used to determine potential cleavage fragments from a protein sequence (with or without specifying a protease-specific cleavage rule) that can be used to assign ions in a spectrum or set of spectra loaded into QUDeX-MS. Digest results from QUDeX can be exported and formatted (including adding assignments from tandem MS) to assess sequence coverage using an external tool such as MSTools [56]. Likewise, the estimated deuterium incorporation for analyzed ions in any of the proposed workflows can be exported in a report that includes the estimated mean and standard deviation of incorporation for each ion and the root-mean-squared error determined by comparing the experimental mass of detected peaks in the spectrum with their theoretical values.

For LC-MS experiments, QUDeX-MS will only calculate the deuterium uptake within a given spectrum. Given the acquisition times necessary to achieve isotopic fine structure-quality spectra (and the fast peptide elution times necessary in a bottom-up HDX experiment to prevent back-exchange), the number of scans a given peptide will be in is limited. The user can average scans of ions detected in an LC-MS run and load that as a single spectrum to calculate deuterium uptake. Given the considerable file sizes associated with LC-MS runs at this resolving power, we recommend limiting the *m/z* range of the resulting averaged spectrum to only include a particular ion’s distribution before loading into QUDeX-MS. Alternatively, the user can process individual (i.e. not averaged) scans, including full scans (although we recommend only loading individual isotopic distributions, if possible), across an LC-MS run and interpret scan-to-scan variability in deuterium uptake calculated for a given ion appropriately.

## Results and discussion

### Calculation of deuterium exchange of a molecule of known composition

**Figure 5:**
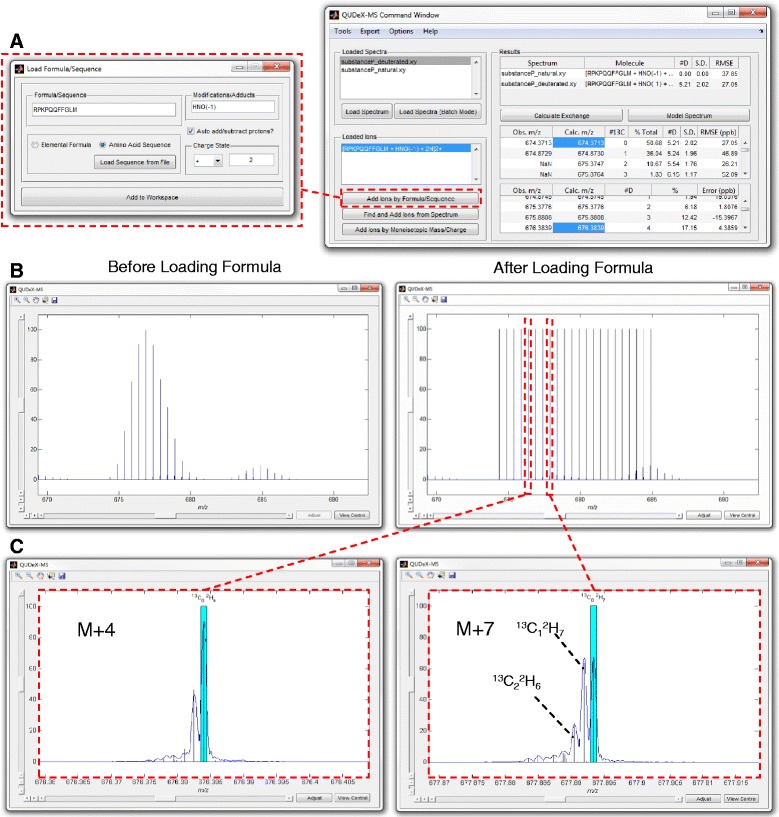
**Determining deuterium incorporation from a molecule of known elemental composition and simulating a theoretical spectrum from calculated deuterium exchange. A**. If the exact elemental formula (or amino acid sequence) of the ion signal to be analyzed is known, this can be provided to QUDeX-MS. If a peptide or protein is being analyzed, in addition to providing an amino acid sequence, the user can specify posttranslational modifications to the amino acid sequence (input as additions and subtractions of elemental atoms), from which the final elemental formula is determined. A specific charge state must also be chosen, which should correspond to a charge state observed in the mass spectrum to be analyzed. **B**. QUDeX-MS *Spectrum Viewer* before and after loading pseudomonoisotopic peak locations (appears as black lines when zoomed out). **C**. Zoomed in view at two fine isotope distributions corresponding to different nominal masses, with the pseudomonoisotopic peak for each distribution highlighted in cyan (black lines in **B**) and calculated theoretical isotopic fine structure based on estimated deuterium incorporation and the specified elemental formula shown as a line spectrum (vertical black lines in **C**). In the analysis of the example spectrum of deuterated substance P, the three major peaks within a fine structure distribution starting from the right moving leftwards are generally the deuterium-associated peaks corresponding to substance P with exactly 0, 1, or 2 ^13^C (and no other natural abundance heavy isotopes) and decreasing number of deuterium, as specified in the panel for the M + 7.

An early step in a typical workflow for HDX MS experiments includes performing tandem mass spectrometry (MS/MS) on a (non-deuterated) digested protein sample in order to identify peptides that will be monitored in later HDX experiments. In this case, where sequence and modification state of peptides has been determined, spectra imported into QUDeX-MS can be analyzed by inputting these known characteristics to determine deuterium exchange (Figure 5A). In example spectra provided with QUDeX-MS, substance P spectra have been acquired both with and without deuterium exchange. The default sequence when loading ions by sequence or formula is set to substance P to help when first learning how to use QUDeX-MS. The substance P analyzed for the example spectra was amidated and the appropriate modification (addition of hydrogen and nitrogen atoms and removal of one oxygen) is also provided as default. A specific charge state must also be chosen, which should correspond to a charge state observed in the mass spectrum to be analyzed. By default, QUDeX-MS assumes the user would like to analyze the protonated form of the input molecule, and thus will adjust the elemental formula by adding the appropriate number of protons (depending on charge state). However, this automatic addition of protons can be disabled, allowing the user to add alternative adducts (such as Na^+^ or K^+^) directly into the elemental formula. Additional modification to a peptide sequence can be performed this way as well.

Once specification of the desired ion to be analyzed is completed, the distribution of *m/z* values corresponding to the deuterium-incorporated forms of the natural abundance monoisotopic molecule are calculated and projected onto the spectrum (Figure 5B). Now that the ion to be analyzed is loaded, the deuterium incorporation can be calculated automatically. In the example of substance P, we see the mean number of incorporated deuterium is estimated from the spectrum to be 5.21 based on the distribution of intensities observed from the deuterium peaks associated with the natural abundance monoisotopic substance P. This was consistent with the value calculated previously using manual annotation of the spectrum [43].

### Modeling theoretical spectrum from calculated deuterium exchange

Included within QUDeX-MS is a method for simulating an isotopic fine structure line spectrum based on the estimated deuterium incorporation distribution and the specified elemental formula. Using the percent contributions of the pseudomonoisotopic peaks, a set of isotopic fine structure spectra are simulated [57] using a set of isotope abundance parameters with a fixed number of deuterium corresponding to the particular pseudomonoisotopic peak. That is, given that the elemental formula for amidated substance P is C_63_H_98_N_18_O_13_S, the [M + 2H]^2+^ distribution based on the population of substance P with no deuterium exchange is modeled using the formula C_63_H_100_N_18_O_13_S and accepted natural isotope abundances. In turn, the [M + 2H]^2+^ distributions for substance P with exactly 1, 2, and 3 deuterium exchanged are modeled using the formulae C_63_DH_99_N_18_O_13_S, C_63_D_2_H_98_N_18_O_13_S, and C_63_D_3_H_97_N_18_O_13_S, and so forth. These distributions are then added together, weighted relative to one another based on their percent contribution estimated from the experimental spectrum. This composite spectrum is projected onto the experimental spectrum (Figure 5C). In the analysis of the example spectrum of deuterated substance P, the three major peaks in fine structure distributions starting from the right moving leftwards are generally the deuterium-associated peaks corresponding to substance P with exactly 0, 1, or 2 ^13^C (and no other natural abundance heavy isotopes).

### Calculation of deuterium exchange from spectra without specifying chemical formula a priori

**Figure 6:**
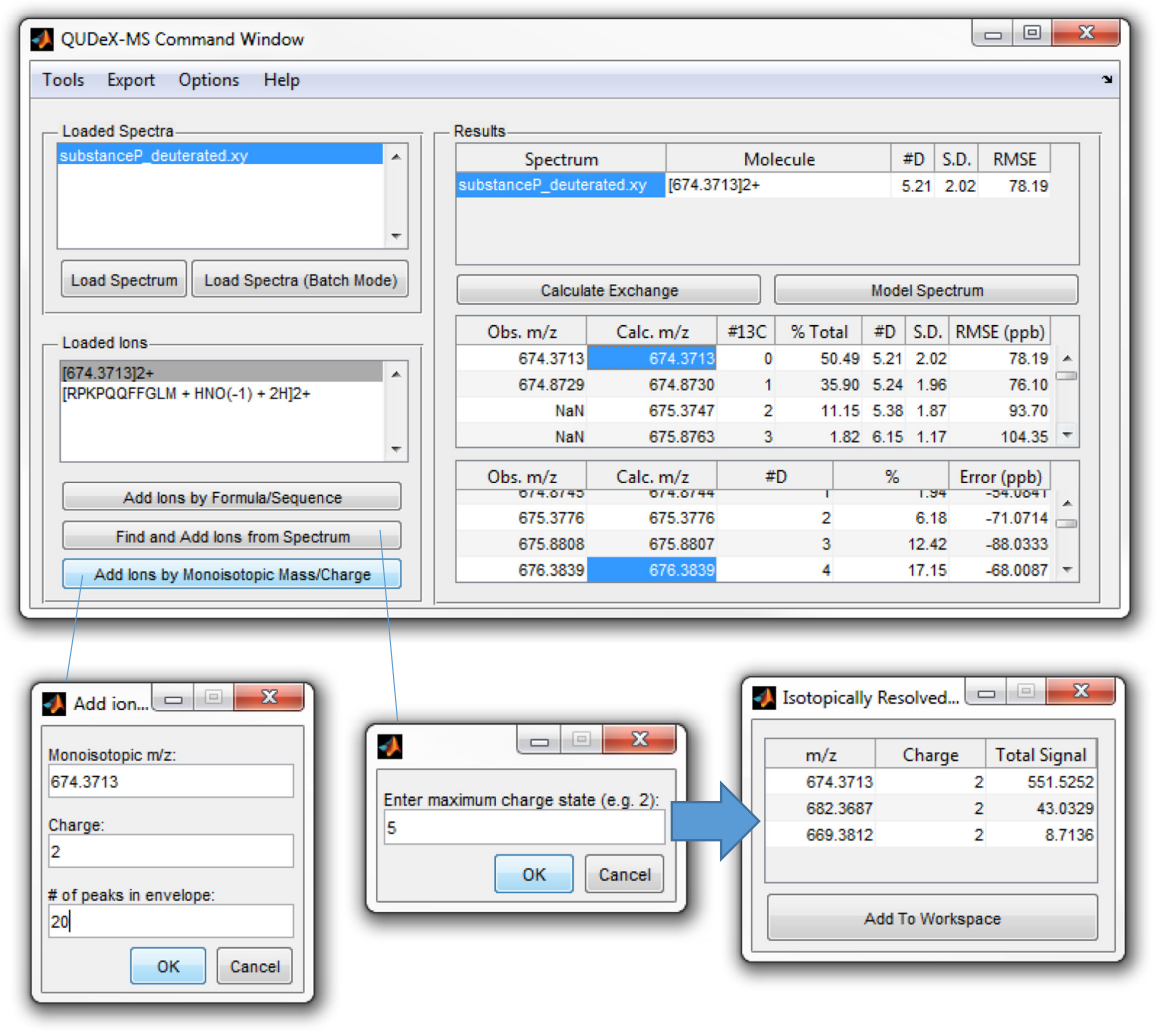
**Determining deuterium incorporation from a molecule of unknown composition.** If the elemental composition of an ion signal being analyzed is not known, or if the user does not want to specify it a priori, deuterium incorporation can be estimated by detecting isotopically resolved ion signals in the spectrum within QUDeX-MS, or by simply specifying a monoisotopic mass from which to calculate a deuterium-associated *m/z* distribution.

Assuming i) a charge state can be determined from an ion signal and ii) the monoisotopic peak in a particular isotopic distribution can be accurately assigned, the *m/z*’s of the deuterium-associated pseudomonoisotopic peaks can be calculated as these are offset by a constant distance determined by the charge and the mass of deuterium. Thus, deuterium incorporation can be estimated for many molecules without a priori knowledge of formula or the spectrum of the undeuterated species. The user can directly specify a monoisotopic *m/z* and charge state to examine, or all isotopically resolved distributions detectable in the spectra can be analyzed (Figure 6). This could be applied to estimating exchange of ions in complex samples of unidentified material such as natural organic matter, as well as studying proteins with unspecified posttranslational modifications or sequence variations. However, it is important to make sure the monoisotopic *m/z*, even if it is not detected, is properly specified in order to correctly estimate deuterium exchange.

### Identification of digest peptides in a deuterated sample from a reference protein sequence

**Figure 7:**
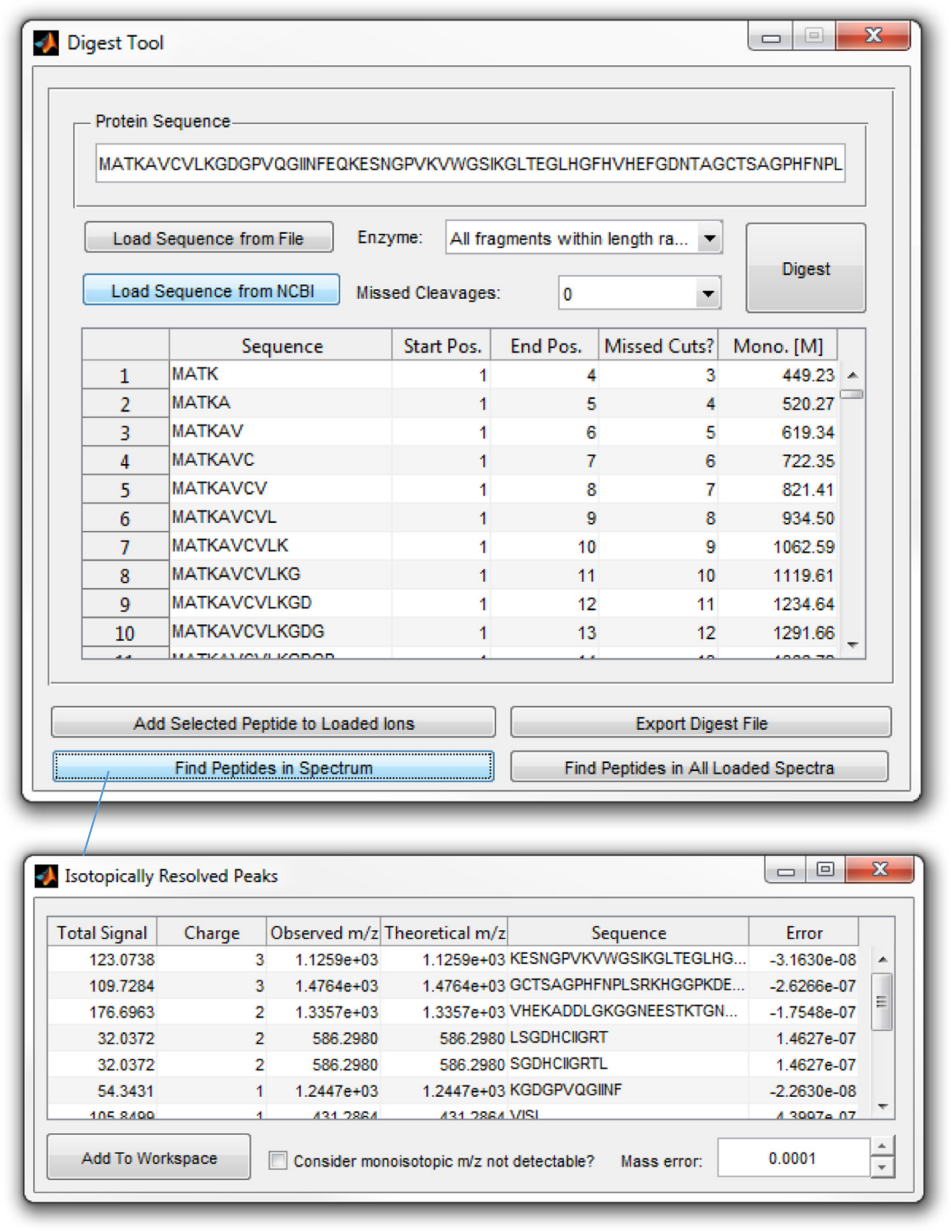
**Digest Tool for automated detection of digest peptides in spectra.** A protein sequence can be loaded and digested *in silico* in QUDeX-MS, and digest fragments can be matched against ions detected in spectra in order to better automate data analysis in a typical HDX experiment. Protein sequences can be pasted into the Protein Sequence field, loaded from a fasta file, or retrieved from NCBI using an appropriate accession. Cleavage specifities for many common proteolytical enzymes are included in QUDeX-MS. Custom cleavage definitions can also be specified, or all possible peptide fragments can be generated for a specified range of peptide lengths. Digest peptides can be added directly to the *Command Window*, further edited individually to add modifications, or can be searched against single or multiple spectra before adding to the *Command Window*. Digest peptides detected in loaded spectra are displayed in a results window (bottom panel). The mass error tolerance can be adjusted in this window, which will update match results automatically. Matching of digest peptides to ions detected in spectra is initially performed based on proper assignment of the monoisotopic mass. However, digest peptides can also be matched against distributions lacking a detectable monoisotopic peak by selecting the “Consider monoisotopic m/z not detectable?” check box. Peptides are then matched to isotopic distributions that fall within *m/z* bins associated with deuterium exchange-associated *m/z*’s within a reasonable distance from the monoisotopic mass (determined by the total number of deuterium exchangeable sites).

In bottom-up HDX experiments studying protein conformation, a proteolytic enzyme is used to digest the protein after the deuterium exchange reaction has been quenched by low pH. To help facilitate use of QUDeX-MS in the context of a typical HDX MS experiment, we have incorporated a “Digest Tool” within the program that allows the user to specify a protein and digest it *in silico*. The protein can be digested using a specified enzyme definition. However, HDX experiments require enzymes such as pepsin that are active at low pH but often cleave with broad specificity, leading to a wide variety of peptides with difficult to predict cleavage sites. High mass accuracy can be particularly useful in this case to help in the accurate assignment of peptide fragments [58,59], although some ambiguities will inevitably remain (such as peptides flanked by the same amino acid on either side). Thus, the QUDeX-MS Digest Tool also allows determining all possible digest fragments of a certain length. Digest peptides can be imported directly into the *Command Window*, edited further to add modifications, or the digest fragments can be matched to peaks found in one or all loaded spectra (Figure 7). We also specifically incorporate the ability to match digest fragments against a deuterated spectrum in the case where the monoisotopic peak is not detected. Digest sequences can be matched against detected isotopic envelopes in a spectrum (even when they are missing a detectable monoisotopic peak) using a combination of (high mass accuracy) mass defect and information regarding the number of exchange sites. This results in the monoisotopic peak being “assigned” (based on the theoretical mass of the peptide) even though it is not detectable in the spectrum. Included with QUDeX-MS is a simulated set of spectra (with resolving power = 1.5 M) from a pepsin digest of deuterated SOD1 based on results we previously observed [9] in order to illustrate how to use the Digest Tool within QUDeX-MS to process fine structure HDX MS data.

### Directions for future development

Currently, QUDeX-MS is specialized for the estimation of deuterium incorporation in the limit where isotopic fine structure is well-resolved, which can be challenging to achieve experimentally. Without well-resolved fine structure, the ultrahigh mass accuracy of FT-ICR instruments can be useful in increasing the number of unambiguous peptide assignments [60]. Indeed, automated and robust procedures have been developed to leverage the ability to resolve closely overlapping distributions (on the order of a few milliDaltons) that can be resolved on FT-ICR instruments [42]. QUDeX-MS offers the opportunity to analyze deuterium-exchange peaks directly, but requires these peaks be resolved in order to function accurately. While resolution of deuterium-associated fine structure is not yet routine, being able to work interchangeably between the resolving power regimes where these peaks are resolved and when they are not would be useful in order to fully utilize datasets.

Applications already available for analyzing and managing data from HDX MS experiments already have many features that enable the visualization and statistical analysis of HDX MS data. Further incorporation of features from these programs would help further bridging the gap between the already existing capabilities of traditional HDX MS and isotopic fine structure HDX MS. Likewise, high mass accuracy afforded on this instrumentation platform can also aid in the unambiguous assignment of peptides, including in the case where only the deuterium-exchanged sample is analyzed, as described here. Both the high mass accuracy and ability to resolve isotopic composition could also be used, combined with HDX data, for the unambiguous identification of the elemental composition of small molecules, as well as their structural elucidation, particularly in complex mixtures such as in natural organic matter or during metabolite profiling. Combining tools used for elemental composition prediction [61-64] with the ability for QUDeX-MS to estimate deuterium incorporation would provide an integrated data analysis environment for data of this type. Indeed, if elemental composition could be determined from accurate mass and/or isotopic composition, small molecule structure prediction combined with HDX data could be used to potentially assign structure as well.

## Conclusions

Isotopic fine structure HDX-MS offers the potential to further increase sequence coverage of proteins being analyzed through high mass accuracy and deconvolution of overlapping ion signals. Many recent advancements in mass spectrometers, including the commercialization of the dynamically harmonized Fourier transform ion cyclotron resonance cell by Ivan Boldin and Eugene Nikolaev [65], have further helped researchers to routinely resolve isotopic fine structure of peptides. Isotopic fine structure HDX-MS has also been demonstrated to be useful in the characterization of molecules in complex mixtures of natural organic matter [66,67]. As previously demonstrated, however, the data analysis workflow for HDX-MS data with resolved isotopic fine structure is distinct. QUDeX-MS we hope will help facilitate the adoption of this technique via streamlining the data analysis process for researchers.

## Availability and requirements

**Project name**: QUDeX-MS

**Project home page**: qudex-ms.sourceforge.net, www.agarlabs.com

**Operating system(s)**: Precompiled versions available for PC (32- and 64-bit) and OSX

**Programming language**: MATLAB

**Other requirements**: None

**License**: FreeBSD

**Any restrictions to use by non-academics**: None

## Abbreviations

MS: Mass spectrometry
FTICR: Fourier transform ion cyclotron resonance
HDX: Hydrogen/deuterium exchange

## References

[1] Yi Q, Baker D (1996). Direct evidence for a two-state protein unfolding transition from hydrogen-deuterium exchange, mass spectrometry, and NMR. Protein Sci.

[2] Engen JR, Wales TE, Chen SG, Marzluff EM, Hassell KM, Weis DD, Smithgall TE (2013). Partial cooperative unfolding in proteins as observed by hydrogen exchange mass spectrometry. Int Rev Phys Chem.

[3] Khanal A, Pan Y, Brown LS, Konermann L (2012). Pulsed hydrogen/deuterium exchange mass spectrometry for time-resolved membrane protein folding studies. J Mass Spectrom.

[4] Eghiaian F, Daubenfeld T, Quenet Y, van Audenhaege M, Bouin AP, van der Rest G, Grosclaude J, Rezaei H (2007). Diversity in prion protein oligomerization pathways results from domain expansion as revealed by hydrogen/deuterium exchange and disulfide linkage. Proc Natl Acad Sci U S A.

[5] Hodkinson JP, Radford SE, Ashcroft AE (2012). The role of conformational flexibility in beta(2)-microglobulin amyloid fibril formation at neutral pH. Rapid Commun Mass Spectrom.

[6] Sitkiewicz E, Tarnowski K, Poznanski J, Kulma M, Dadlez M: **Oligomerization interface of RAGE receptor revealed by MS-monitored hydrogen deuterium exchange.** *PLoS One* 2013, **8**(10):e76353. 10.1371/journal.pone.0076353 PMC3788119 24098480

[7] Smirnovas V, Kim JI, Lu XJ, Atarashi R, Caughey B, Surewicz WK (2009). Distinct structures of scrapie prion protein (PrPSc)-seeded versus spontaneous recombinant prion protein fibrils revealed by hydrogen/deuterium exchange. J Biol Chem.

[8] Pan J, Han J, Borchers CH, Konermann L (2012). Structure and dynamics of small soluble abeta(1–40) oligomers studied by top-down hydrogen exchange mass spectrometry. Biochemistry.

[9] Molnar KS, Karabacak NM, Johnson JL, Wang Q, Tiwari A, Hayward LJ, Coales SJ, Hamuro Y, Agar JN (2009). A common property of amyotrophic lateral sclerosis-associated variants: destabilization of the copper/zinc superoxide dismutase electrostatic loop. J Biol Chem.

[10] Tang L, Coales SJ, Morrow JA, Edmunds T, Hamuro Y (2012). Characterization of the N370S mutant of glucocerebrosidase by hydrogen/deuterium exchange mass spectrometry. Chem BioChem.

[11] Rose RJ, van Berkel PHC, van den Bremer ETJ, Labrijn AF, Vink T, Schuurman J, Heck AJR, Parren PWHI (2013). Mutation of Y407 in the CH3 domain dramatically alters glycosylation and structure of human IgG. MAbs.

[12] Chetty PS, Ohshiro M, Saito H, Dhanasekaran P, Lund-Katz S, Mayne L, Englander W, Phillips MC (2012). Effects of the Iowa and Milano mutations on apolipoprotein a-I structure and dynamics determined by hydrogen exchange and mass spectrometry. Biochemistry.

[13] Hamuro Y, Wong L, Shaffer J, Kim JS, Stranz DD, Jennings PA, Woods VL, Adams JA (2002). Phosphorylation driven motions in the COOH-terminal Src kinase, Csk, revealed through enhanced hydrogen-deuterium exchange and mass spectrometry (DXMS). J Mol Biol.

[14] Bobst CE, Thomas JJ, Salinas PA, Savickas P, Kaltashov IA (2010). Impact of oxidation on protein therapeutics: Conformational dynamics of intact and oxidized acid-beta-glucocerebrosidase at near-physiological pH. Protein Sci.

[15] Mitra G, Muralidharan M, Narayanan S, Pinto J, Srinivasan K, Mandal AK (2012). Glutathionylation induced structural changes in oxy human hemoglobin analyzed by backbone amide hydrogen/deuterium exchange and MALDI-mass spectrometry. Bioconjug Chem.

[16] Balchin D, Stoychev SH, Dirr HW (2013). S-nitrosation destabilizes glutathione transferase p 1–1. Biochemistry.

[17] Pan JX, Borchers CH (2013). Top-down structural analysis of posttranslationally modified proteins by Fourier transform ion cyclotron resonance-MS with hydrogen/deuterium exchange and electron capture dissociation. Proteomics.

[18] Chalmers MJ, Busby SA, Pascal BD, West GM, Griffin PR (2011). Differential hydrogen/deuterium exchange mass spectrometry analysis of protein-ligand interactions. Expert Rev Proteomics.

[19] Lanman J, Prevelige PE (2004). High-sensitivity mass spectrometry for imaging subunit interactions: hydrogen/deuterium exchange. Curr Opin Struct Biol.

[20] Xiao H, Kaltashov IA, Eyles SJ (2003). Indirect assessment of small hydrophobic ligand binding to a model protein using a combination of ESI MS and HDX/ESI MS. J Am Soc Mass Spectrom.

[21] Tropak MB, Kornhaber GJ, Rigat BA, Maegawa GH, Buttner JD, Blanchard JE, Murphy C, Tuske SJ, Coales SJ, Hamuro Y, Brown ED, Mahuran DJ (2008). Identification of pharmacological chaperones for Gaucher disease and characterization of their effects on beta-glucocerebrosidase by hydrogen/deuterium exchange mass spectrometry. Chem BioChem.

[22] Chalmers MJ, Busby SA, Pascal BD, He Y, Hendrickson CL, Marshall AG, Griffin PR (2006). Probing protein ligand interactions by automated hydrogen/deuterium exchange mass spectrometry. Anal Chem.

[23] Weinreb PH, Li S, Gao SX, Liu T, Pepinsky RB, Caravella JA, Lee JH, Woods VL (2012). Dynamic structural changes are observed upon collagen and metal ion binding to the integrin alpha 1 I domain. J Biol Chem.

[24] Trelle MB, Dupont DM, Madsen JB, Andreasen PA, Jorgensen TJD (2014). Dissecting the effect of RNA aptamer binding on the dynamics of plasminogen activator inhibitor 1 using hydrogen/deuterium exchange mass spectrometry. ACS Chem Biol.

[25] Frego L, Gautschi E, Martin L, Davidson W (2006). The determination of high-affinity protein/inhibitor binding constants by electrospray ionization hydrogen/deuterium exchange mass spectrometry. Rapid Commun Mass Spectrom.

[26] Kaltashov IA, Bobst CE, Abzalimov RR, Berkowitz SA, Houde D (2010). Conformation and dynamics of biopharmaceuticals: transition of mass spectrometry-based tools from academe to industry. J Am Soc Mass Spectrom.

[27] Kaltashov IA, Bobst CE, Abzalimov RR, Wang G, Baykal B, Wang S (2012). Advances and challenges in analytical characterization of biotechnology products: mass spectrometry-based approaches to study properties and behavior of protein therapeutics. Biotechnol Adv.

[28] Bobst CE, Abzalimov RR, Houde D, Kloczewiak M, Mhatre R, Berkowitz SA, Kaltashov IA (2008). Detection and characterization of altered conformations of protein pharmaceuticals using complementary mass spectrometry-based approaches. Anal Chem.

[29] Engen JR (2009). Analysis of protein conformation and dynamics by hydrogen/deuterium exchange MS. Anal Chem.

[30] Mandell JG, Falick AM, Komives EA (1998). Measurement of amide hydrogen exchange by MALDI-TOF mass spectrometry. Anal Chem.

[31] Nazabal A, Laguerre M, Schmitter JM (2003). Hydrogen/deuterium exchange on yeast ATPase supramolecular protein complex analyzed at high sensitivity by MALDI mass spectrometry. J Am Soc Mass Spectrom.

[32] Liu S, Liu L, Uzuner U, Zhou X, Gu M, Shi W, Zhang Y, Dai SY, Yuan JS: **HDX-analyzer: a novel package for statistical analysis of protein structure dynamics.** *BMC Bioinformatics* 2011, **12**(Suppl 1):S43. 10.1186/1471-2105-12-S1-S43 PMC3044300 21342575

[33] Weis DD, Engen JR, Kass IJ (2006). Semi-automated data processing of hydrogen exchange mass spectra using HX-Express. J Am Soc Mass Spectrom.

[34] Pascal BD, Chalmers MJ, Busby SA, Mader CC, Southern MR, Tsinoremas NF, Griffin PR: **The Deuterator: software for the determination of backbone amide deuterium levels from H/D exchange MS data.** *BMC Bioinformatics* 2007, **8:**156. 10.1186/1471-2105-8-156 PMC1876250 17506883

[35] Pascal BD, Chalmers MJ, Busby SA, Griffin PR (2009). HD desktop: an integrated platform for the analysis and visualization of H/D exchange data. J Am Soc Mass Spectrom.

[36] Hotchko M, Anand GS, Komives EA, Ten Eyck LF (2006). Automated extraction of backbone deuteration levels from amide H/2H mass spectrometry experiments. Protein Sci.

[37] Slysz GW, Baker CA, Bozsa BM, Dang A, Percy AJ, Bennett M, Schriemer DC: **Hydra: software for tailored processing of H/D exchange data from MS or tandem MS analyses.** *BMC Bioinformatics* 2009, **10:**162. 10.1186/1471-2105-10-162 PMC2696453 19473537

[38] Nikamanon P, Pun E, Chou W, Koter MD, Gershon PD: **“TOF2H”: a precision toolbox for rapid, high density/high coverage hydrogen-deuterium exchange mass spectrometry via an LC-MALDI approach, covering the data pipeline from spectral acquisition to HDX rate analysis.** *BMC Bioinformatics* 2008, **9:**387. 10.1186/1471-2105-9-387 PMC2561049 18803853

[39] Kreshuk A, Stankiewicz M, Lou XH, Kirchner M, Hamprecht FA, Mayer MP (2011). Automated detection and analysis of bimodal isotope peak distributions in H/D exchange mass spectrometry using HeXicon. Int J Mass Spectrom.

[40] Lindner R, Lou X, Reinstein J, Shoeman RL, Hamprecht FA, Winkler A (2014). Hexicon 2: automated processing of hydrogen-deuterium exchange mass spectrometry data with improved deuteration distribution estimation. J Am Soc Mass Spectrom.

[41] Guttman M, Weis DD, Engen JR, Lee KK (2013). Analysis of overlapped and noisy hydrogen/deuterium exchange mass spectra. J Am Soc Mass Spectrom.

[42] Kazazic S, Zhang HM, Schaub TM, Emmett MR, Hendrickson CL, Blakney GT, Marshall AG (2010). Automated data reduction for hydrogen/deuterium exchange experiments, enabled by high-resolution Fourier transform ion cyclotron resonance mass spectrometry. J Am Soc Mass Spectrom.

[43] Liu Q, Easterling ML, Agar JN (2014). Resolving isotopic fine structure to detect and quantify natural abundance- and hydrogen/deuterium exchange-derived isotopomers. Anal Chem.

[44] Marshall AG, Comisarow MB, Parisod G (1979). Relaxation and spectral-line shape in Fourier-transform ion resonance spectroscopy. J Chem Phys.

[45] Marshall AG, Hendrickson CL, Jackson GS (1998). Fourier transform ion cyclotron resonance mass spectrometry: A primer. Mass Spectrom Rev.

[46] Hsu CS, Hendrickson CL, Rodgers RP, McKenna AM, Marshall AG (2011). Petroleomics: advanced molecular probe for petroleum heavy ends. J Mass Spectrom.

[47] Xian F, Hendrickson CL, Blakney GT, Beu SC, Marshall AG (2010). Automated broadband phase correction of Fourier transform ion cyclotron resonance mass spectra. Anal Chem.

[48] Qi YL, Thompson CJ, Van Orden SL, O’Connor PB (2011). Phase correction of Fourier transform ion cyclotron resonance mass spectra using MatLab. J Am Soc Mass Spectrom.

[49] Qi YL, Barrow MP, Li HL, Meier JE, Van Orden SL, Thompson CJ, O’Connor PB (2012). Absorption-mode: the next generation of Fourier transform mass spectra. Anal Chem.

[50] Kilgour DPA, Wills R, Qi YL, O’Connor PB (2013). Autophaser: an algorithm for automated generation of absorption mode spectra for FT-ICR MS. Anal Chem.

[51] Denisov E, Damoc E, Lange O, Makarov A (2012). Orbitrap mass spectrometry with resolving powers above 1,000,000. Int J Mass Spectrom.

[52] Michalski A, Damoc E, Lange O, Denisov E, Nolting D, Muller M, Viner R, Schwartz J, Remes P, Belford M, Dunyach JJ, Cox J, Horning S, Mann M, Makarov A: **Ultra high resolution linear ion trap Orbitrap mass spectrometer (Orbitrap Elite) facilitates top down LC MS/MS and versatile peptide fragmentation modes.** *Mol Cell Proteomics* 2012, **11**(3):O111.01369. 10.1074/mcp.O111.013698 PMC3316736 22159718

[53] Barclay VJ, Bonner RF, Hamilton IP (1997). Application of wavelet transforms to experimental spectra: smoothing, denoising, and data set compression. Anal Chem.

[54] Donoho DL, Johnstone IM (1995). Adapting to unknown smoothness via wavelet shrinkage. J Am Stat Assoc.

[55] Coombes KR, Tsavachidis S, Morris JS, Baggerly KA, Hung MC, Kuerer HM (2005). Improved peak detection and quantification of mass spectrometry data acquired from surface-enhanced laser desorption and ionization by denoising spectra with the undecimated discrete wavelet transform. Proteomics.

[56] Kavan D, Man P (2011). MSTools – Web based application for visualization and presentation of HXMS data. Int J Mass Spectrom.

[57] Rockwood AL, VanOrden SL, Smith RD (1996). Ultrahigh resolution isotope distribution calculations. Rapid Commun Mass Spectrom.

[58] Lam TT, Lanman JK, Emmett MR, Hendrickson CL, Marshall AG, Prevelige PE (2002). Mapping of protein:protein contact surfaces by hydrogen/deuterium exchange, followed by on-line high-performance liquid chromatography-electrospray ionization Fourier-transform ion-cyclotron-resonance mass analysis. J Chromatogr A.

[59] Zhang HM, Kazazic S, Schaub TM, Tipton JD, Emmett MR, Marshall AG (2008). Enhanced digestion efficiency, peptide ionization efficiency, and sequence resolution for protein hydrogen/deuterium exchange monitored by Fourier transform ion cyclotron resonance mass spectrometry. Anal Chem.

[60] Okawa S, Fischer B, Krijgsveld J (2013). Properties of isotope patterns and their utility for peptide identification in large-scale proteomic experiments. Rapid Commun Mass Spectrom.

[61] Pluskal T, Uehara T, Yanagida M (2012). Highly accurate chemical formula prediction tool utilizing high-resolution mass spectra, MS/MS fragmentation, heuristic rules, and isotope pattern matching. Anal Chem.

[62] Patiny L, Borel A (2013). ChemCalc: a building block for tomorrow’s chemical infrastructure. J Chem Inf Model.

[63] Kind T, Fiehn O: **Seven golden rules for heuristic filtering of molecular formulas obtained by accurate mass spectrometry.** *BMC Bioinformatics* 2007, **8:**105. 10.1186/1471-2105-8-105 PMC1851972 17389044

[64] Kind T, Fiehn O: **Metabolomic database annotations via query of elemental compositions: mass accuracy is insufficient even at less than 1 ppm.** *BMC Bioinformatics* 2006, **7:**234. 10.1186/1471-2105-7-234 PMC1464138 16646969

[65] Boldin IA, Nikolaev EN (2011). Fourier transform ion cyclotron resonance cell with dynamic harmonization of the electric field in the whole volume by shaping of the excitation and detection electrode assembly. Rapid Commun Mass Spectrom.

[66] Cho Y, Ahmed A, Kim S (2013). Application of atmospheric pressure photo ionization hydrogen/deuterium exchange high-resolution mass spectrometry for the molecular level speciation of nitrogen compounds in heavy crude oils. Anal Chem.

[67] Kostyukevich Y, Kononikhin A, Popov I, Kharybin O, Perminova I, Konstantinov A, Nikolaev E (2013). Enumeration of labile hydrogens in natural organic matter by use of hydrogen/deuterium exchange Fourier transform ion cyclotron resonance mass spectrometry. Anal Chem.

